# Role of Infection Prevention and Control Practices in Preventing COVID-19 Reinfection Among Healthcare Workers: A Single-Centre Experience From North India

**DOI:** 10.7759/cureus.96662

**Published:** 2025-11-12

**Authors:** Rakesh Gupta, Ritu Sharma, Suprakash Mandal, Ravi Kumar, Abhishek Bharti, Kausar Neyaz

**Affiliations:** 1 Pediatrics, Government Institute of Medical Sciences, Greater Noida, IND; 2 Obstetrics and Gynecology, Government Institute of Medical Sciences, Greater Noida, IND; 3 Community Medicine, Government Institute of Medical Sciences, Greater Noida, IND; 4 Medical Research Unit, Government Institute of Medical Sciences, Greater Noida, IND; 5 Preventive and Social Medicine, Government Institute of Medical Sciences, Greater Noida, IND

**Keywords:** covid-19, eye-protection, gloves, hand-hygiene, healthcare workers, infection prevention and control measures, mask, personal protective equipment, sars-cov2

## Abstract

Introduction: The emergence of COVID-19 surges, exposing healthcare workers (HCWs) to the risk of COVID-19 reinfection, raises the alarm and prompts the search for the most effective infection prevention and control (IPC) practices to promote and sustain their rationalised use. We aimed to assess the role of individual IPC practices in preventing COVID-19 reinfection among HCWs.

Methods: This was a hospital-based, cross-sectional study conducted during the pandemic that included consenting HCWs involved in COVID care with RTPCR-confirmed COVID-19 infection. We used a self-developed, pre-tested, interviewer-administered, semi-structured questionnaire to collect clinico-demographic details and attitudes towards IPC practices. The responses of HCWs with a single infection and those with reinfection were compared.

Results: Resident doctors exhibited higher odds of reinfection (OR = 14.11; 95% CI: 3.58-55.59; p < 0.001); HCWs assigned to OT/ICUs or laboratories demonstrated significantly lower odds of reinfection (OR = 0.30; 95% CI: 0.11-0.84; p = 0.021); those who were on duty within the hospital faced significant risk of reinfection (OR = 3.54; 95% CI: 1.19-10.51; p = 0.023). Both partially and fully vaccinated HCWs had lower odds of reinfection compared to unvaccinated individuals. Random Forest analysis showed that among the IPC practices, mask use provided the greatest protection against reinfection, followed by the use of sterile gloves.

Conclusion: Strengthening and rationalising IPC practices are critical to reducing the risk of COVID-19 reinfection among HCWs.

## Introduction

The COVID-19 pandemic has been responsible for immense morbidity and mortality worldwide. Transmitted by the SARS-CoV-2 virus, the pandemic has unfolded in three waves with the emergence of new variants of concern (VOCs), exposing healthcare workers (HCWs) to the risk of reinfections. Important VOCs include Alpha, Beta, Delta, and Omicron [1]. Though the end of the pandemic was declared on 5th May 2023, regional surges continue to occur [2]. A recent surge occurred due to new variants under monitoring: NB.1.8.1 and LF.7 [3].

Immunity against COVID-19 can be achieved through prior infection or vaccination. Vaccination, the primary preventive strategy, had reduced COVID-19 infections and their severity, but, on its own, was unable to control transmission. The duration of immunity and efficacy offered by the vaccine were variable, with higher protection against severe disease and against certain VOCs, such as the delta variant [4]. Infection prevention and control (IPC) practices have a proven role in containing infections both in hospitals and in the community. Social distancing, though an effective preventive strategy, is not feasible in hospital settings for vulnerable HCWs; thus, personal protective equipment (PPE) is recommended for HCWs by the WHO, which includes a mask, gloves, eye protection, a gown, maintaining hand hygiene, and a safe distance. Using proper PPE has a statistically insignificant protective effect against COVID-19 infection compared with no PPE (OR = 0.52; 95% CI: 0.13-2.12; low-certainty evidence) [5]. To support the rational use of PPE kits in meagre-resource settings, WHO continued to update the initial version of the evidence-based guidelines as new evidence emerged and released the seventh version of the IPC guideline for COVID-19 in December 2023 [6-8]. However, evidence of the efficacy of IPC measures, both individually and in different combinations, in COVID-19 infection remains limited. Moreover, the recent surge raised alarm about identifying independent factors associated with COVID-19 reinfection and the appropriate PPE measures.

Consequently, we aimed to assess the role of individual IPC practices in preventing COVID-19 reinfection among HCWs and to identify independent risk factors associated with COVID-19 reinfection among HCWs.

## Materials and methods

Study design

This was a hospital-based, cross-sectional study conducted during the COVID-19 pandemic from 25 July 2022 to 31 December 2022.

Study settings

The study was conducted at a tertiary care hospital in Western Uttar Pradesh (UP), India. The institute is the only government tertiary care institute providing care to a large patient population from Western UP and neighbouring states. The hospital was converted to a dedicated COVID-19 care centre during the pandemic.

Study population

The study population comprised all HCWs involved in patient care at the hospital during the pandemic, from 1 April 2020 to 31 May 2022.

Inclusion criteria

The study included HCWs who had RTPCR-confirmed COVID-19 infection. For reinfection, patients who met the epidemiological reinfection diagnostic criteria, i.e., those with positive RTPCR test results >90 days after prior infection [9], were included.

Exclusion criteria

COVID-19 infections not confirmed by RTPCR and RTPCR-positive in <90 days of prior infection (for reinfected cases) were excluded from the study.

Sample size and sampling technique

The list of HCWs with RTPCR-confirmed COVID-19 infections during the study period (1 April 2020 to 31 May 2022) was obtained from the molecular laboratory. We conveniently included all the consenting HCWs who met the eligibility criteria for infection and reinfection.

Data collection procedure

For the interview, we used a self-developed, pre-tested, interviewer-administered, semi-structured questionnaire, validated by a panel of experts at the institute. The questionnaire comprised three sections: the first section dealt with the basic sociodemographic details. The second section was related to COVID-19 vaccination status at the time of initial infection and reinfection. HCWs were classified in three groups: (1) unvaccinated; (2) partially vaccinated (received one vaccination dose only/two doses with the second dose <15 days); and (3) fully vaccinated (two doses with the second dose >15 days/three doses). The last section captured data on the perception of HCWs towards the cause of their infection and IPC practices followed by HCWs that included masks, gloves, eye protection, gowns, maintaining hand hygiene, and safe distance.

For IPC practice we used a 5-point Likert scale to measure and score the responses: always (practice followed >95% of the time; 5 points), most of the time (practice followed >50% but <95% of the time; 4 points), sometimes (practice followed >20% but <50% of the time; 3 points), rarely (practice followed <20% of the time; 2 points), or never (1 point).

Those who followed an IPC measure "always" or "most of the time" were considered compliant with that measure.

Statistical analysis

Data were entered into Microsoft Excel (Microsoft Corporation, Redmond, Washington) and analysed using the IBM SPSS Statistics for Windows, Version 28 (Released 2021; IBM Corp., Armonk, New York). Categorical variables were presented as frequencies and proportions (%) and continuous variables as means (SD) or medians (IQR). Compliance scores for each IPC measure were summed, and a mean (SD) was calculated. Unpaired t-test/Mann-Whitney U test was used to compare the continuous variables with normal and skewed distribution, and the chi-square test was used to compare the categorical variables. Binary and multivariable logistic regression were used to assess the strength of association among the predictor variables. The predictor variables with p-values up to 0.20 in the binary regression were included in the multivariable model. An odds ratio with a 95% confidence interval was used to report the point estimate of the regression result. Apart from that, the significance of infection prevention variables was assessed utilising a random forest model, which quantified the contribution of each predictor through two distinct metrics: Mean Decrease in Accuracy (MDA) and Mean Decrease in Gini (MDG). The model was trained on IPC variables, including mask, gloves, eye shield, gown, distancing, and hygiene. These metrics facilitated the identification of variables that exerted the most substantial influence on the predictive accuracy of the model and the configuration of the decision tree. The level of significance was considered at 0.05.

## Results

**Figure 1:**
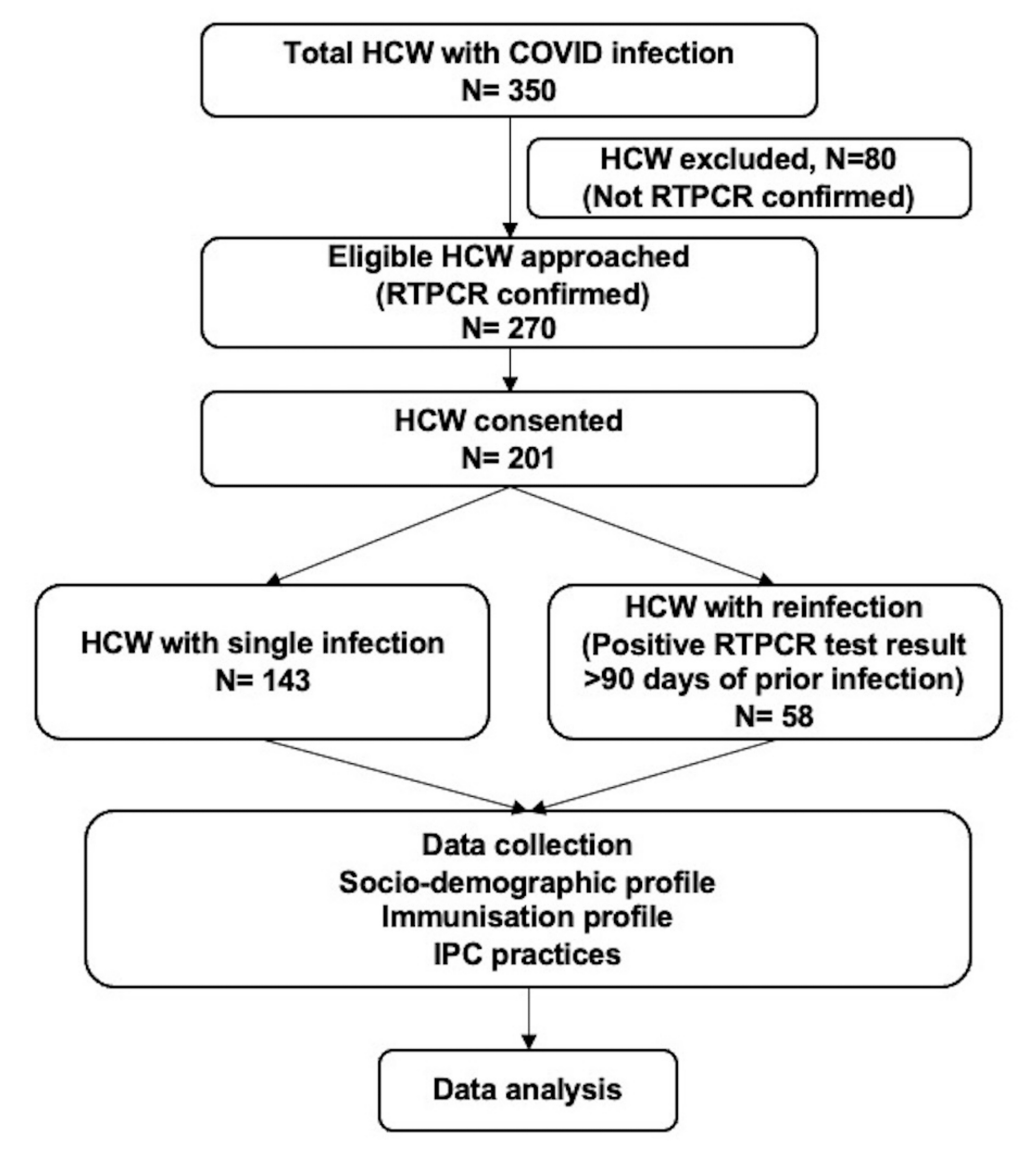
Flow chart of the study design HCW: healthcare workers; IPC: infection prevention and control

The total number of HCWs with COVID-19 infection in the specified period was 350, and 80 HCWs were excluded as infection was not confirmed by RTPCR. Of the 270 HCWs with RTPCR-confirmed COVID-19 infection who were approached for the survey, 201 consented to participate, yielding a response rate of 74.4%. Among 201 HCWs, 143 (71.1%) had a single episode of infection, while 58 (28.9%) had reinfection. Responses in the two groups were compared (Figure 1).

Demographic profile of HCWs

The mean age of HCWs with single-episode infection was 29.97 ± 6.64 years, which was comparable to that of the reinfection group (30.29 ± 6.95 years). The majority of infected HCWs in both groups were aged 25-40 years (162, 80.6%). Male HCWs predominated in both groups, with no statistically significant difference (p-value = 0.194). The two groups differed significantly with respect to the field of work: nurses were outnumbered in the single-infection group (64 (44.8%), p < 0.001), while the resident doctors were outnumbered in the reinfection group (17, 29.3%). Most of the HCWs in the single-episode infection and the reinfection group had work experience between two and five years (93 (65.0%) and 32 (55.2%), respectively). As far as the area of work was concerned, in the single-infection group, the majority of HCWs were working in maximum aerosol-generating areas (OT/ICU/laboratory) (62, 43.4%), while the majority of HCWs in the reinfection group were working in OPD at the time of reinfection (24, 41.4%). In the single-episode infection group, the most common blood group was B+ve (51, 35.66%), while in the reinfection group, it was B-ve (23, 39.7%). Overall, only 14 (7%) HCWs had associated co-morbidities, the most common being hypothyroidism, and 5 (2.5%) HCWs reported persistence of deranged blood sugar values for variable periods after COVID-19 infection, with no statistically significant difference between the two groups (Table 1).

**Table 1:** Comparison of the basic sociodemographic profile of the participants between the two groups The data were presented as frequency (percentage) for categorical variables. Categorical variables were analysed using the chi-square test/Fisher's exact test. The relevant test statistic and p-value for each variable are indicated in the table. A p-value of <0.05 was considered statistically significant. n: number of patients in the group; HCWs: healthcare workers

Variables	Categories	Total HCWs With COVID-19 Infection n = 201 n (%)	HCWs Without Reinfection n= 143 n (%)	HCWs With Reinfection n = 58 n (%)	Test Statistic, Test Name, p-value
Age (years)	<25	33 (16.4)	18 (12.6)	5 (8.6)	χ² = 0.84, chi-square, p = 0.658
	25–40	162 (80.59)	113 (79)	49 (84.4)	
	>40	16 (7.9)	12 (8.4)	4 (6.8)	
Sex	Male	115 (57.2)	80 (55.9)	35 (60.3)	χ² = 0.17, chi-square, p = 0.679
	Female	86 (42.8)	63 (44.1)	23 (39.7)	
Field of work	Housekeeping/attendants	30 (14.9)	24 (16.8)	6 (10.3)	χ² = 23.17, chi-square, p < 0.001
	Resident doctors	26 (12.9)	9 (6.3)	17 (29.3)	
	Consultant doctors	13 (6.5)	8 (5.6)	5 (8.6)	
	Nurses	78 (38.8)	64 (44.8)	14 (24.1)	
	Paramedical	54 (26.9)	38 (26.6)	16 (27.6)	
Total experience (years)	<2	22 (10.9)	13 (9.1)	9 (15.5)	χ² = 2.38, chi-square, p = 0.304
	2–5	125 (62.2)	93 (65.0)	32 (55.2)	
	>5	54 (26.9)	37 (25.8)	17 (29.3)	
Area of work	OPD	68 (33.8)	44 (30.8)	24 (41.4)	χ² = 4.42, chi-square, p = 0.110
	Ward	55 (27.4)	37 (25.8)	18 (31.0)	
	OT/ICU/lab	78 (38.8)	62 (43.4)	16 (27.6)	
Presence of co-morbidities	Yes	14 (7.0)	8 (5.6)	6 (10.3)	χ² = 0.80, chi-square, p = 0.372
	No	187 (93.0)	135 (94.4)	52 (89.7)	
Deranged blood sugars after infection	Yes	5 (2.5)	3 (2.1)	2 (3.5)	Fisher's exact, p = 0.577
	No	196 (97.5)	140 (97.9)	56 (96.6)	

COVID-19 immunisation profile of HCWs

There was no statistically significant difference between the two groups with respect to COVID-19 immunisation profiles at the time of infection and reinfection. At the time of initial infection, only 52 (36.4%) HCWs in the single-episode infection group and 16 (27.6%) HCWs in the reinfection group were fully vaccinated, while the majority of HCWs in both groups were unvaccinated. At the time of reinfection, nearly all HCWs were fully vaccinated. Overall, among those vaccinated (partial/fully), the median duration (IQR) between the last vaccination dose and initial infection was 57 (42-81) days with no significant difference (Table 2).

**Table 2:** Comparison of the COVID-19 immunisation profile and perception of the participants towards the cause of COVID-19 infection in two groups The variables were presented as frequencies (percentages), except for the duration between the last vaccination and the first infection, which was presented as median (IQR). Categorical variables were analysed using the chi-square test or the Mann-Whitney U test. The relevant test statistic and p-value for each variable are indicated in the table. A p-value of <0.05 was considered statistically significant. n: number of patients in the group; HCWs: healthcare workers

Variables	Category	Total HCWs With COVID-19 Infection n = 201 n (%)	HCWs Without Reinfection n = 143 n (%)	HCWs With Reinfection n = 58 n (%)	Test Statistic, Test Name, p-value
COVID-19 immunisation profile					
Status of vaccination at the time of first infection	Unvaccinated	113 (56.2)	78 (54.5)	35 (60.3)	χ² = 1.55, chi-square, p = 0.460
	Partially vaccinated (one dose only/2 doses with second dose < 15 days)	20 (10.0)	13 (9.1)	7 (12.1)	
	Fully vaccinated (2 doses with second dose >15 days/3 doses)	68 (33.8)	52 (36.4)	16 (27.6)	
Duration between the last vaccination and first infection (days)	Median (IQR)	57 (42–81)	57.5 (42–85.5)	55.5 (43–70)	z= 0.332, Mann-Whitney U, p= 740
Fully vaccinated status at time of reinfection	Yes	196 (97.5)	140 (97.9)	56 (96.6)	χ² = 0.003, chi-square, p = 0.811
	No	5 (2.5)	3 (2.1)	2 (3.5)	
Perception of the participants towards the cause of COVID-19 infection					
What do you think from where you acquired the infection?	On duty at hospital	104 (51.7)	55 (38.5)	49 (84.5)	χ² = 33.18, chi-square, p = 0.001
	At home	97 (48.3)	88 (61.5)	9 (15.5)	
What do you think was the risk factor for reinfection?	Contact	150 (74.6)	101 (70.6)	49 (84.5)	χ² = 9.84, chi-square, p = 0.041
	Breach in IPC measures	28 (13.9)	26 (18.2)	2 (3.5)	
	Travel	12 (6)	10 (6.9)	2 (3.5)	
	Not taken prophylaxis	11 (5.5)	6 (4.2)	5 (8.6)	

Perception of HCWs towards the cause of infection

A significantly higher proportion of HCWs in the single-infection group reported infection at home (88, 61.5%), whereas in the reinfection group, they reported infection while on duty at the hospital (49, 84.5%). The opinion survey revealed that the majority of HCWs in the two groups attributed close "contact" as the most likely cause of their infection, followed by breaches in IPC measures, with a significant difference in perception between the two groups (p-value = 0.041) (Table 2).

Attitude of HCWs towards IPC measures

HCWs in single-episode infection and reinfection groups were most compliant for maintaining hand hygiene and mask usage, with over 97% of HCWs in each group reporting compliance for hand hygiene ("always"/"most of the time") (139 (97.2%) vs. 57 (98.2%), respectively). More than 75% reported compliance using masks (109 (76.2%) vs. 49 (84.5%), respectively). The mean scores for these practices were also high (4.8 for hand hygiene; >4 for mask usage) with no significant difference between the two groups.

In contrast, compliance with surgical glove use was low in the single-episode infection and reinfection groups, with around 40% of HCWs in each group reporting compliance (62 (43.4%) vs. 22 (37.9%), respectively). The mean scores were slightly higher among the single-episode infection group than the reinfected group, suggesting marginally better compliance in the former group (3.4 (+0.9) vs. 3.2 (+1.1), respectively).

Again, maintaining a 2-metre distance was inconsistently practised: 52 (36.4%) HCWs in the single-episode infection group and 25 (43.1%) HCWs in the reinfection group were compliant in maintaining a safe distance, though the overall mean scores were moderate (3.4 (+0.8) vs. 3.5 (+0.7), respectively).

HCWs in both groups had low compliance with the use of medical gowns. The majority of HCWs in the single-episode infection and reinfection groups admitted using medical gowns sometimes only (61 (42.7%) vs. 26 (42.8%), respectively), and an almost equal number admitted rarely or never using the gowns (64 (44.75%) vs. 21 (36.2%), respectively). The mean scores were below 3.

Protective shields/glasses had the lowest compliance, with over 70% of HCWs in both groups reporting "rarely" or "never" using them (114 (79.7%) vs. 43 (74.1%) in the single-episode infection and reinfection groups, respectively), and the mean scores were below 2.

Overall, there was no significant difference between the two groups with respect to the attitude of HCWs towards different IPC measures (Tables 3, 4).

**Table 3:** Attitude of HCWs in two groups towards Infection Prevention and Control (IPC) measures (based on a 5-point Likert scale) Attitude of HCWs in the two groups towards Infection Prevention and Control (IPC) measures (based on a 5-point Likert scale). The variables were presented as frequency (percentage).

Variables	Always (5) n (%)	Most of the Time (4) n (%)	Sometimes (3) n (%)	Rarely (2) n (%)	Never (1) n (%)
Mask					
Total HCW with COVID-19 infection (N = 201)	87 (43.3)	71 (35.3)	38 (18.9)	5 (2.5)	0 (0.0)
HCW with no COVID-19 reinfection (N = 143)	62 (43.4)	47 (32.9)	29 (20.3)	5 (3.5)	0 (0.0)
HCW with COVID-19 reinfection (N = 58)	25 (43.1)	24 (41.4)	9 (15.5)	0 (0.0)	0 (0.0)
Surgical gloves					
Total HCW with COVID-19 infection (N = 201)	25 (12.4)	59 (29.4)	81 (40.3)	26 (12.9)	10 (5.0)
HCW with no COVID-19 reinfection (N = 143)	20 (14.0)	42 (29.4)	60 (42.0)	15 (10.5)	6 (4.2)
HCW with COVID-19 reinfection (N = 58)	5 (8.6)	17 (29.3)	21 (36.2)	11 (19.0)	4 (6.9)
Eye protection – shield/glasses					
Total HCW with COVID-19 infection (N = 201)	4 (2.0)	4 (2.0)	36 (17.9)	78 (38.8)	79 (39.3)
HCW with no COVID-19 reinfection (N = 143)	2 (1.4)	1 (0.7)	26 (18.2)	60 (42.0)	54 (37.8)
HCW with COVID-19 reinfection (N = 58)	2 (3.4)	3 (5.2)	10 (17.2)	18 (31.0)	25 (43.1)
Medical gown					
Total HCW with COVID-19 infection (N = 201)	6 (3.0)	23 (11.4)	87 (43.3)	46 (22.9)	39 (19.4)
HCW with no COVID-19 reinfection (N = 143)	4 (2.8)	14 (9.8)	61 (42.7)	37 (25.9)	27 (18.9)
HCW with COVID-19 reinfection (N = 58)	2 (3.4)	9 (15.5)	26 (44.8)	9 (15.5)	12 (20.7)
Maintain a 2-metre distance					
Total HCW with COVID-19 infection (N = 201)	16 (8.0)	61 (30.3)	109 (54.2)	15 (7.5)	0 (0.0)
HCW with no COVID-19 reinfection (N = 143)	11 (7.7)	41 (28.7)	78 (54.5)	13 (9.1)	0 (0.0)
HCW with COVID-19 reinfection (N = 58)	5 (8.6)	20 (34.5)	31 (53.4)	2 (3.4)	0 (0.0)
Maintain hand hygiene					
Total HCW with COVID-19 infection (N = 201)	169 (84.1)	27 (13.4)	4 (2.0)	1 (0.5)	0 (0.0)
HCW with no COVID-19 reinfection (N = 143)	121 (84.6)	18 (12.6)	3 (2.1)	1 (0.7)	0 (0.0)
HCW with COVID-19 reinfection (N = 58)	48 (82.8)	9 (15.5)	1 (1.7)	0 (0.0)	0 (0.0)

**Table 4:** Comparison of the mean score of the attitude of HCW in two groups towards Infection Prevention and Control (IPC) measures (based on responses on a 5-point Likert scale, with a maximum score of 5) The data were represented as mean (SD). The choice of test (unpaired t-test or Mann-Whitney U test) was based on the data distribution. The relevant test statistic and p-value for each variable were indicated in the table. A p-value of <0.05 was considered statistically significant.

Variables	HCWs With No COVID-19 Reinfection, n = 143	HCWs With COVID-19 Reinfection, n = 58	Test Name, Test Statistic, p-value
	Mean (SD)	Mean (SD)	
Mask	4.3 (0.8)	4.1 (0.8)	Mann-Whitney U, z=-0.629, p=0.53
Surgical gloves	3.4 (0.9)	3.2 (1.1)	Unpaired t-test, t=1.56, p=0.118
Eye protection – shield/glasses	1.9 (0.8)	1.8 (0.9)	Mann-Whitney U, z=-0.044, p=0.964
Medical gown	2.5 (1.0)	2.6 (1.0)	Mann-Whitney U, z=-0.747, p=0.455
Maintain a 2-m distance	3.4 (0.8)	3.5 (0.7)	Unpaired t-test, t=-1.5, p=0.134
Maintain hand hygiene	4.8 (0.4)	4.8 (0.5)	Mann-Whitney U, z=0.434, p=0.664

Logistic regression

Compared with nursing officers, resident doctors exhibited higher odds of reinfection (OR = 14.11; 95% CI: 3.58-55.59; p < 0.001), indicating a substantially greater risk. HCWs assigned to operating theatres, ICUs, or laboratories demonstrated significantly lower odds of reinfection relative to their counterparts in outpatient departments (OR = 0.30; 95% CI: 0.11-0.84; p = 0.021). Furthermore, individuals who were on duty within the hospital faced a significantly elevated risk of reinfection (OR = 3.54; 95% CI: 1.19-10.51; p = 0.023).

Among IPC practices, glove usage and physical distancing had variable degrees of association, but none of them showed statistically significant associations with reinfection.

Based on the regression results, both partially and fully vaccinated individuals had lower odds ratios as compared to unvaccinated individuals (OR = 0.46; 95% CI: 0.13-1.68; and OR = 0.64; 95% CI: 0.26-1.55, respectively). Although the risk of reinfection was 23% lower in partially vaccinated HCWs and 55% lower in fully vaccinated HCWs compared to unvaccinated HCWs, the results indicate no significant difference between the unvaccinated and the partially or fully vaccinated groups (Table 5).

**Table 5:** Results of multivariable logistic regression for the reinfection status across different predictor variables Variables with p-values < 0.2 in the binary regression were included in the multivariable model. The level of significance was set at p < 0.05.

Predictor	Beta	aOR	95% CI	p-value
Field of work in the health sector (Ref: Nursing)				
Resident	2.65	14.11	3.58–55.59	<0.001
Consultant	1.28	3.61	0.69–19	0.129
Paramedics	0.61	1.84	0.57–5.96	0.31
Housekeeping	-0.25	0.78	0.22–2.76	0.702
Total experience in years (Ref: <2 years)				
2–5 years	-0.46	0.63	0.17–2.38	0.498
≥5 years	-0.26	0.77	0.18–3.39	0.733
Area of work (Ref: OPD)				
OT/ICU/Lab	-1.21	0.3	0.11–0.84	0.021
Ward	0.13	1.14	0.37–3.45	0.822
Have you received BCG vaccination during COVID-19? (Ref: No)				
Yes	0.09	1.09	0.38–3.16	0.869
Where were you at the time of reinfection? (Ref: At home)				
On duty at hospital	1.26	3.54	1.19–10.51	0.023
Reason for reinfection (Ref: Travel)				
Breach in IPC measures	-1.59	0.2	0.02–2.44	0.209
Contact	-0.04	0.96	0.13–7.23	0.971
Not taken prophylaxis	0.99	2.7	0.24–29.96	0.419
Do you use surgical gloves? (Ref: Always)				
Most of the times	0.42	1.52	0.35–6.63	0.576
Sometimes	-0.02	0.98	0.24–3.97	0.974
Rarely	0.78	2.17	0.44–10.68	0.34
Never	0.71	2.04	0.28–14.82	0.482
Do you maintain 2 m distance? (Ref: Always)				
Most of the times	-0.08	0.92	0.22–3.93	0.912
Sometimes	-0.11	0.9	0.21–3.81	0.885
Rarely	-1.29	0.28	0.02–3.46	0.318
Never	Insufficient data			
Vaccination status (Ref: Unvaccinated)				
Fully vaccinated	-0.45	0.64	0.26–1.55	0.318
Partially vaccinated	-0.77	0.46	0.13–1.68	0.241

Random forest classifier

**Figure 2:**
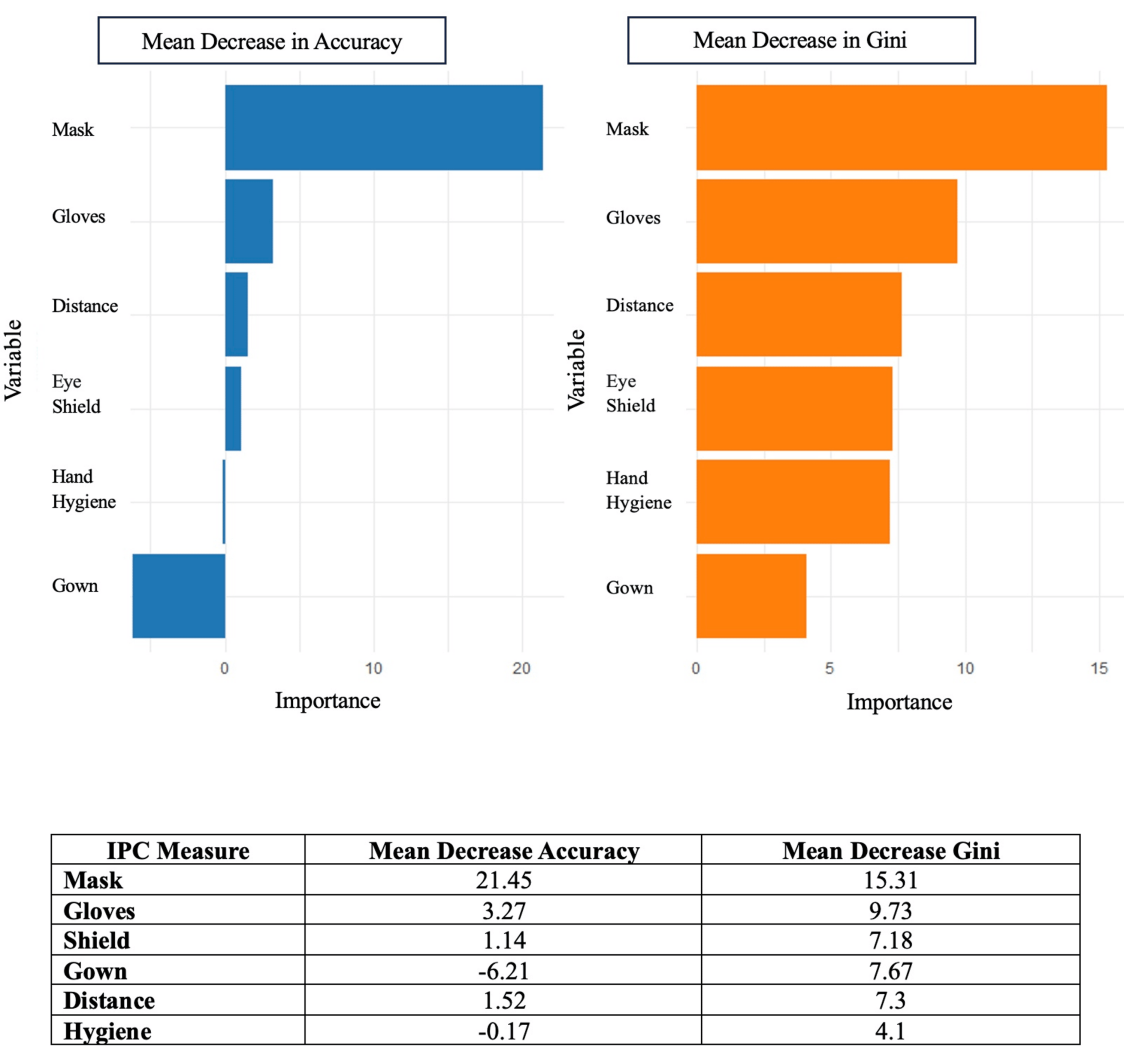
Variable importance plot from a random forest model for the different infection prevention practices IPC: infection prevention and control

Random forest analysis showed that among the IPC measures, mask use was the strongest predictor of protection from reinfection, with the highest MDA (21.4) and the MDG impurity (15.3). Gloves also contributed moderately (MDA = 3.3; MDG = 9.7). Shield use, gowns, distancing, and hand hygiene had lower or slightly negative importance scores, indicating limited added protection (Figure 2).

## Discussion

Our study aimed to explore the factors contributing to the risk of reinfection in a hospital setting and the effects of various infection prevention measures.

The majority of infected HCWs in both groups in our study were aged 25-40 years (80.6%). This is consistent with a study by Korkusuz et al., which found that 73.1% of HCWs were under 40 years of age [10]. This may be due to the involvement of mainly young HCWs in COVID care. Nurses predominated in our study, accounting for 38.8% of infections. Korkusuz et al. also observed similar findings, with 41.9% of HCWs being nurses [10]. This may be due to the fact that the maximum number of staff involved in providing direct COVID care, with prolonged contact time, was nursing staff. Similar to our study, Korkusuz et al. observed that only a few HCWs had co-morbidities (17.9%), and this observation aligns with the expert consensus to keep HCWs with co-morbidities away from direct COVID care, considering the increased risk of severe infection in them [10].

On analysing risk factors for COVID-19 reinfection, we found that resident doctors were significantly more susceptible compared to nursing staff, which may be potentially attributable to their risk-taking behaviour and involvement in procedural activities. Furthermore, HCWs assigned to OT/ICU/laboratories demonstrated a significantly lower risk compared to those working in OPD. The results are consistent with other studies and are likely indicative of more rigorous compliance with IPC protocols in these high-aerosol-generating environments [10,11]. Being on duty in the hospital also significantly increased the risk of reinfection, highlighting occupational exposure as a critical contributing factor.

We observed that partially and fully vaccinated individuals had lower odds ratios for COVID-19 reinfection than unvaccinated individuals, though the difference was not statistically significant. Hedberg et al. also reported that compared with unvaccinated individuals, the aOR for developing persistent post-COVID-19 condition (PCC) was reduced in vaccinated individuals; the risk reduces further with an increase in the number of doses received: aOR 0.81 (95% CI, 0.59-1.10) for one dose, aOR 0.42 (95% CI, 0.35-0.52) for two doses, and aOR 0.37 (95% CI, 0.27-0.52) for three doses [12]. Taherian et al. reported a significant reduction in infection, hospitalisation, and mortality rates (per 1000 population) in fully vaccinated individuals compared with unvaccinated individuals (3.9, 1.08, and 0.09, respectively, vs. 69.7, 12.1, and 1.04, respectively) [13]. We can conclude from the above results that vaccination provides variable immunity and reduces the risk and severity of COVID-19 infection.

Ensuring compliance with preventive interventions remains challenging but essential to contain infection. In our study, we found that HCWs in both single-infection and reinfection groups were most compliant with maintaining hand hygiene (>97%). Mustafa et al. also reported high compliance for hand hygiene [14], whereas Korkusuz et al. reported low compliance [10]. A longitudinal, cross-sectional study conducted in ICU settings in the pre-pandemic and during-pandemic periods reported a significant positive effect of the COVID-19 pandemic on hand hygiene practices and emphasised the need for strict compliance in all situations and for its sustainability [15].

We also observed high compliance for mask usage in both groups (>75%). Similar high compliance with mask use was observed in other studies [10,14,16].

Similar to our findings, Khubchandani et al. also reported low compliance for glove usage (30%) [16]. In contrast, Korkusuz et al. reported significantly higher infected HCWs (77.8%) using gloves always as recommended (p = 0.012) [10]. Mustafa et al. also found that HCWs were compliant with glove use [14].

We observed very low compliance for the use of medical gowns and eye shields/glasses, with mean scores for the latter below 2 in both groups. Similar and statistically lower compliance among infected HCWs was observed by Korkusuz et al. for eye protection (p = 0.01) and by Mustafa et al. for gowns [10,14].

Random forest analysis in our study showed that, among the IPC measures, mask use was the strongest predictor of reinfection, followed by surgical glove use.

Mask use by HCWs had also been shown to reduce the risk of COVID-19 infection by 70% in other studies [17]. In a rapid review and meta-analysis of the effectiveness of PPE for HCWs during the COVID-19 pandemic, it was reported that face masks offer significant protection against COVID-19 infection (OR = 0.16; 95% CI: 0.04-0.58; moderate-grade evidence).

In a 2011 Cochrane review of IPC measures to reduce the spread of respiratory viruses, Jefferson et al. reported that masks were the most effective preventive measure across settings and populations [18]. However, a Cochrane review in 2023, with the same lead author, focussing on respiratory viral infections during epidemic and non-epidemic influenza seasons (from 2003 onwards), the H1N1 pandemic, and the COVID-19 pandemic, reported little or no effect of masks on COVID-19 outcomes (RR 0.95, 95% CI 0.84-1.09) in community settings and little or no effect of N95/P2 respirators compared to medical/surgical masks (RR 1.10, 95% CI 0.90-1.34) in hospital settings [19]. Another review by the same lead author found that masks alone, without other measures, were not recommended due to a lack of evidence [20].

The evidence-based IPC guideline for COVID-19, December 2023, by WHO recommends that, while providing care to a patient with suspected or confirmed COVID-19, HCWs should wear a respirator or medical mask in addition to other PPE [7].

Schoberer et al. found no protective effect of wearing gloves against COVID-19 infection among HCWs (OR = 1.09; 95% CI: 0.25-4.19; low-grade evidence) [5].

In another meta-analysis including eight heterogeneous studies, a significant decrease in the incidence of healthcare-associated infections was observed with universal gloving alone (IRR 0.77; 95% CI 0.67-0.89); however, no such association was observed with intervention bundles that included universal gloving also (IRR 0.95; 95% CI 0.86-1.05). The study concluded that, given the small protective effect of universal gloving against healthcare-associated infections, it may be judiciously reserved for high-risk settings [21].

Hand hygiene has consistently been recognised as a prime preventive practice; however, we did not find an association between hand hygiene and reinfection, which may be due to the universal practice of hand hygiene in both groups. A meta-analysis including two studies with a small sample size of 144 reported a statistically non-significant protective effect of hand hygiene (OR = 0.43; 95% CI: 0.11-1.64; low-grade evidence) [5]. A Cochrane review (2023) explored the role of hand hygiene and reported an 11% relative reduction of respiratory illness (RR 0.89, 95% CI 0.83-0.94; low-certainty evidence), which, though, was statistically insignificant [19]. It has been found that the HCWs who are non-compliant with the use of gloves and hand hygiene have an increased risk of infection subsequent to hand-to-face contact. Recognising the limited protection gloves provide, WHO has emphasised that hand hygiene cannot be ignored even when gloves are used [22]. The results of a meta-analysis also revealed no effect (OR = 1.07; 95% CI 0.43-2.64) on infection associated with the gown use [5]. We also could not find any association between reinfection and wearing gowns.

A Cochrane review emphasised the need for large, high-quality randomised controlled trials (RCTs) to assess the effectiveness of IPC practices across various settings and the impact of compliance on effectiveness [19]. Recently, a qualitative study conducted during the pre-COVID-19 pandemic and during the pandemic periods concluded that HCWs demanded a practical and easy-to-follow approach for preventive measures with proven added value [23].

Our study provides evidence that masks followed by gloves offer the maximum protection to HCWs against COVID-19 reinfection; hence, it suggests tailored IPC interventions based on profession and healthcare setting.

Limitations and strengths

A single-centre study with a small sample size is our limitation that may affect the generalisability of the results. Recall bias is another limitation, but since the information collected was related to standard protocols and recent events, any recall bias should be minimal.

Strengths include the inclusion of HCWs who had similar exposure and practised the same protocols and who were infected with COVID-19 either once or multiple times in an attempt to investigate the protective role of individual IPC practices. Robust statistical analysis is another strength of our study.

## Conclusions

Strengthening rationalised IPC practices is critical to foster pandemic preparedness and reduce infection transmission to and from HCWs. The reflections by HCWs in our study regarding IPC practices provided evidence that mask and glove use provided maximum protection against COVID-19 reinfection. However, high-quality, multicentric research studies are required to confirm the certainty of evidence and to comment on the efficacy of different combinations of PPE.

## Appendices

Case Record Form

Title: Assessing the Role of Infection Prevention and Control Practices in Preventing COVID-19 Reinfection Among Healthcare Workers - A single centre Experience from North India

Date _______________                                                  Enrolment Code __________________

**Table 6:** Basic socio-demographic profile

Variables	Categories	Responses (put the tick mark), Remarks
Age (years)	<25	
	25-40	
	> 40	
Sex	Male	
	Female	
Field of work	Housekeeping/ attendants	
	Resident doctors	
	Consultant doctors	
	Nurses	
	Paramedical	
Total experience (years)	<2	
	2-5	
	>5	
Area of work	OPD	
	Ward	
	OT/ ICU/ Lab	
Presence of Co-morbidities	Yes	
	No	
Deranged blood sugars after infection	Yes	
	No	

**Table 7:** COVID-19 Immunization profile of the participants

	COVID-19 immunization profile	Definition	Response
What was the status of COVID vaccination at time of first infection?	Unvaccinated	Not vaccinated yet	
	Partially vaccinated	Received 1 dose only Received 2 doses with second dose < 15 days	
	Fully vaccinated	Received 2 doses with second dose >15 days Received 3doses	
What was the duration between the last vaccination and first infection (days)?			
Were you fully vaccinated at time of re-infection? (received 2 doses with second dose >15 days/ 3 doses)	Yes		
	No		

**Table 8:** Perception of the participants towards the cause of COVID-19 infection

	Perception of the participants towards the cause of COVID-19 infection	Response
What do you think from where had you acquired infection?	On duty at hospital	
	At home	
What do you think was the risk factor for re-infection?	Contact	
	Breach in IPC measures	
	Travel	
	Not taken Prophylaxis	

**Table 9:** Attitude of HCW towards Infection Prevention and Control (IPC) measures based on 5-point Likert scale Definition: Always (practice followed > 95% of the times; 5 points) Most of the times (practice followed > 50% but < 95% of the times; 4 points) Sometimes (practice followed > 20% but < 50% of the times; 3 points) Rarely (practice followed < 20% of times; 2 points) Never (1 point)

Statement	Always (5)	Most of times (4)	Sometimes (3)	Rarely (2)	Never (1)
How often are you using Mask as IPC measure?					
How often are you using Surgical gloves as IPC measure?					
How often are you using Protective eye-shield / glasses as IPC measure?					
How often are you using medical gown as IPC measure?					
How often are you maintaining 2m distance as IPC measure?					
How often are you maintaining hand hygiene as IPC measure?					

CRF Status: Complete/Incomplete (Reason: _________________________)

Signature of PI:
